# Effectiveness and safety of Liuhedan for treating cellulitis: A protocol for systematic review and meta-analysis

**DOI:** 10.1097/MD.0000000000026118

**Published:** 2021-06-04

**Authors:** Na Li, Li Zhang, Pan Pan, Tao Cheng, Shu-Yun Xu

**Affiliations:** a Hematology Department, Cheng Du Shang Jin Nan Fu Hospital; b Department of Emergency Medicine and Laboratory of Emergency Medicine, West China Hospital, Sichuan University, Chengdu, Sichuan, China.

**Keywords:** cellulitis, duration of hospital stays, Liuhedan, meta-analysis, prognosis, systematic review, traditional Chinese medicine

## Abstract

**Background:**

Liuhedan is a famous traditional Chinese Medicine formula used to treat cellulitis in China. However, there are no systematic reviews for the evidence and the therapeutic effectiveness and safety of Liuhedan for treating cellulitis. The aim of this study is to summarize previous evidence, assessing the efficacy and safety of Liuhedan treating cellulitis.

**Methods:**

We will search the EMBASE, WANFANG DATA, Web of Knowledge, CNKI, PubMed, ClinicalTrials.gov, and Cochrane Library from inception to June 30, 2021 to retrieve relevant studies using the search strategy: (“Liuhedan” OR “Liuhe Pill” OR “Liu-He-Dan”) AND (“cellulitis” OR “phlegmon” OR “skin and soft tissue infection” OR “skin tissue infection” OR “soft tissue infection”). Two authors independently judged study eligibility and extracted data. Heterogeneity will be examined by computing the *Q* statistic and *I*^2^ statistic.

**Results:**

This study assessed the efficiency and safety of Liuhedan for treating cellulitis.

**Conclusions:**

This study will provide reliable evidence-based evidence for the clinical application of Liuhedan for treating cellulitis.

**Ethics and dissemination:**

Ethical approval is unnecessary as this protocol is only for systematic review and does not involve privacy data. The findings of this study will be disseminated electronically through a peer-review publication or presented at a relevant conference.

## 1 Introduction

Cellulitis is an infection of the deep dermis and subcutaneous tissue, presenting with expanding erythema, warmth, tenderness, and swelling.^[1–3]^ Cellulitis is a common global health burden, with >650,000 admissions per year in the United States alone, costing 3.7 billion dollars per year. Although common, it often can be a therapeutic challenge.^[3,4]^ Therefore, it is necessary to improve the managements of patients with cellulitis. Previous researches showed that Liuhedan can reduce the inflammatory index^[5,6]^ and it has been used to treat cellulitis in China for many years. Despite this, there are no published systematic reviews and meta-analyses exploring the effectiveness and safety of Liuhedan treating cellulitis. Therefore, we conducted this systematic review and meta-analysis to clarify the efficacy and safety of Liuhedan for treating cellulitis.

## 2 Methods and analysis

### 2.1 Registration

This protocol of systematic review and meta-analysis is based on the Preferred Reporting Items for Systematic Reviews and meta-analysis Protocols (PRISMA-P) statement guidelines. And the protocol has been registered on International Prospective Register of Systematic Reviews database. The registration number was INPLASY202110049.

### 2.2 Eligibility criteria

The inclusion criteria for the study will include: age ≥18 years old and diagnosis of cellulitis; the patients with cellulitis was divided into 2 groups (treated with Liuhedan or without Liuhedan); conference abstracts were only included when they provided adequate relevant information for assessment.

### 2.3 Searching strategy

**Figure 1:**
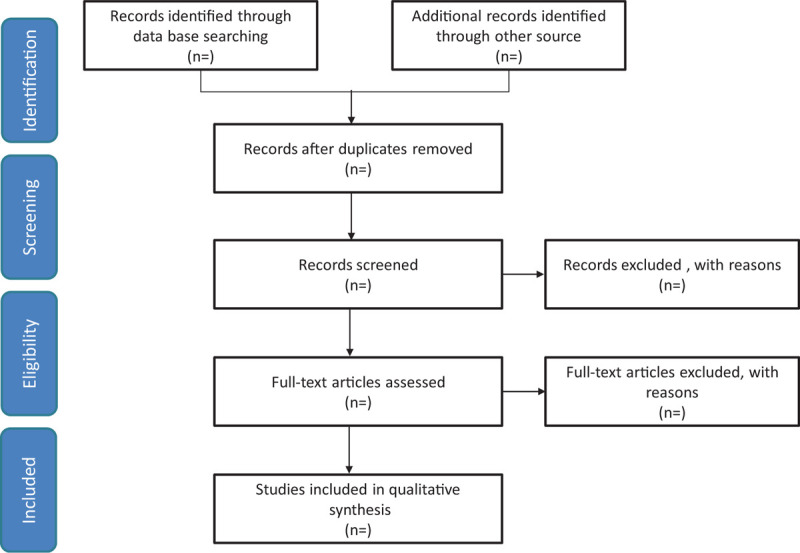
A flow diagram demonstrating the search strategy and study selection process for this study.

We will search the EMBASE, WANFANG DATA, Web of Knowledge, CNKI, PubMed, ClinicalTrials.gov, and Cochrane Library from inception to June 30, 2021 to retrieve relevant studies using the search strategy: (“Liuhedan” OR “Liuhe Pill” OR “Liu-He-Dan”) AND (“cellulitis” OR “phlegmon” OR “skin and soft tissue infection” OR “skin tissue infection” OR “soft tissue infection”). No language restrictions will be applied. We will also search citations of relevant primary and review. Authors of abstract in the meeting will be further searched in PubMed for potential full articles. To minimize the risk of publication bias, we will conduct a comprehensive search that included strategies to find published and unpublished studies. The research summary of the screening flow chart is shown in Fig. 1.

### 2.4 Data extraction and risk of bias

Two reviewers will be employed the searching strategy respectively, by reading the papers and scoring them according to the QUADAS-2 checklist^[7]^ and Newcastle-Ottawa Quality Assessment Scale^[8]^; disagreement will be settled by a third opinion. Important information will be abstracted from the included articles in a standardized form by 2 reviewers. Important information includes the name of the first author, publication year, type of study, study population, sample size, using of Liuhedan, and outcomes studied. Risk of bias assessment will be carried out according to the Newcastle-Ottawa Scale (NOS) to rate the internal validity of the individual studies, and funnel plots will be constructed to assess the risk of publication bias.

### 2.5 Statistical analysis

All pairwise meta-analytic calculations will be performed with Review Manager software (RevMan) version 5.3 (Cochrane Collaboration). Heterogeneity will be examined by computing the *Q* statistic and *I*^2^ statistic, and presence of reporting bias by visual inspection of funnel plots. Statistical significance was considered when the *P* value <.05.

## 3 Discussion

Cellulitis is a common and expensive problem worldwide, presenting with expanding erythema, warmth, tenderness, and swelling.^[1,3]^ It often complicate inflammatory response syndrome triggered by bacterial toxins and inflammatory mediators.^[3]^ The constituents of Liuhedan include raw rhubarb, raw cortex phellodendri, mint, bletilla, and radix angelicae.^[9,10]^ Liuhedan is able to clear away heat and toxic materials, relive swelling and pain as well as dispel stasis and dampness^[11]^ and it could reduce the inflammatory index by anti-inflammatory and immunomodulating, which may be the theoretical basis for the treatment of cellulitis. Combination of Chinese herb medicine Liuhedan and western medicine has been used to treat cellulitis in China for many years. However, effectiveness and safety of Liuhedan for treating cellulitis are controversial. Therefore, we designed the systematic review and meta-analysis protocol by using the latest data to test the effectiveness and safety of Liuhedan treating cellulitis. The results of our review will be reported strictly following the PRISMA criteria. We hope that our findings will find more rigorous medical evidence for the application of Liuhedan treating cellulitis, thus providing a reference for clinical practice.

## Acknowledgments

The authors would like to acknowledge the participants and their families for taking part in the study.

## Author contributions

**Conceptualization:** Na Li, Li Zhang, Tao Cheng, Pan Pan, Shu-Yun Xu.

**Data curation:** Na Li, Li Zhang, Pan Pan, Shu-Yun Xu.

**Formal analysis:** Na Li, Tao Cheng, Pan Pan.

**Funding acquisition:** Shu-Yun Xu.

**Investigation:** Na Li, Li Zhang, Tao Cheng.

**Methodology:** Na Li, Li Zhang, Pan Pan.

**Project administration:** Na Li, Tao Cheng.

**Resources:** Na Li, Li Zhang, Shu-Yun Xu.

**Software:** Na Li, Li Zhang, Pan Pan.

**Supervision:** Na Li, Li Zhang, Shu-Yun Xu.

**Validation:** Na Li, Li Zhang, Tao Cheng, Pan Pan.

**Visualization:** Na Li, Li Zhang.

**Writing – original draft:** Na Li, Pan Pan, Shu-Yun Xu.

**Writing – review & editing:** Tao Cheng, Shu-Yun Xu.
